# Biological Roles of the *Podospora anserina* Mitochondrial Lon Protease and the Importance of Its N-Domain

**DOI:** 10.1371/journal.pone.0038138

**Published:** 2012-05-31

**Authors:** Céline Adam, Marguerite Picard, Michelle Déquard-Chablat, Carole H. Sellem, Sylvie Hermann-Le Denmat, Véronique Contamine

**Affiliations:** 1 Univ Paris-Sud, Institut de Génétique et Microbiologie, UMR 8621, Orsay, France; 2 CNRS, Orsay, France; 3 CNRS, Centre de Génétique Moléculaire, UPR 3404, Gif-sur-Yvette, France; 4 Ecole Normale Supérieure, Paris, France; Seoul National University, Republic of Korea

## Abstract

Mitochondria have their own ATP-dependent proteases that maintain the functional state of the organelle. All multicellular eukaryotes, including filamentous fungi, possess the same set of mitochondrial proteases, unlike in unicellular yeasts, where ClpXP, one of the two matricial proteases, is absent. Despite the presence of ClpXP in the filamentous fungus *Podospora anserina*, deletion of the gene encoding the other matricial protease, *PaLon1*, leads to lethality at high and low temperatures, indicating that PaLON1 plays a main role in protein quality control. Under normal physiological conditions, the *PaLon1* deletion is viable but decreases life span. *PaLon1* deletion also leads to defects in two steps during development, ascospore germination and sexual reproduction, which suggests that PaLON1 ensures important regulatory functions during fungal development. Mitochondrial Lon proteases are composed of a central ATPase domain flanked by a large non-catalytic N-domain and a C-terminal protease domain. We found that three mutations in the N-domain of PaLON1 affected fungal life cycle, PaLON1 protein expression and mitochondrial proteolytic activity, which reveals the functional importance of the N-domain of the mitochondrial Lon protease. All *PaLon1* mutations affected the C-terminal part of the N-domain. Considering that the C-terminal part is predicted to have an α helical arrangement in which the number, length and position of the helices are conserved with the solved structure of its bacterial homologs, we propose that this all-helical structure participates in Lon substrate interaction.

## Introduction

Mitochondria are essential organelles in all eukaryotes and have many functions aside from the production of energy. To ensure proper function, mitochondria possess their own set of ATP-dependent proteases, which are homologous to bacterial proteases; these include two metalloproteases of the FtsH bacterial family, which are lodged in the mitochondrial inner membrane and two serine proteases, ClpXP and Lon, which are found in the mitochondrial matrix. The i-AAA and m-AAA metalloproteases have been implicated in the biogenesis of respiratory complexes and have active sites located in the intermembrane space and matrix, respectively [1] (for review). The ClpXP protease differs from the other proteases; firstly, its ATPase (ClpX) and protease (ClpP) domains are divided into two polypeptides, and secondly, ClpP is absent in most yeasts. Not much is known regarding the specific function of ClpXP, and the relationship between Lon and ClpXP remains to be clarified. In mammalian cells, while *ClpP*, but not *Lon*, is transcriptionally upregulated when an aggregated protein accumulates within the mitochondrial matrix [2], [3], the protein levels of the ClpP and Lon proteases appear to decrease or increase together during pathological conditions [4], [5].

Mitochondrial Lon was first identified in humans [6] and in the yeast *Saccharomyces cerevisiae* [7], [8]. Mitochondrial Lon proteins share common properties with their well-characterized bacterial homologs, including the capacity to bind DNA and their essential role in protein quality control by destroying abnormal proteins, such as respiratory chain subunits and metabolic enzymes [9] (for review). By contrast, the regulatory role established for bacterial Lon in controlling the availability of specific proteins has not been documented for mitochondrial Lon. However, mutated Lon has been found to impact plant development [10]. Similar to their bacterial homologs, mitochondrial Lon form homo-oligomeric structures [9] and are composed of an N-domain, followed by a central ATPase domain and a C-terminal protease domain.

Here, we describe the defects associated with the deletion of the *PaLon1* gene, which encodes the mitochondrial Lon protease in the filamentous fungus *Podospora anserina*. In this strict aerobe, the absence of PaLON1 was tolerated, but led to a decrease in life span. PaLON1 was essential in extreme growth conditions (high and low temperatures). We found that PaLON1 was important during ascospore germination and sexual reproduction, two regulated developmental steps. We also characterized the defects associated with three *PaLon1* mutations that affect the N-domain. Two mutations affected ascospore germination and life span to the same extent as the *PaLon1* deletion, but differed in their sensitivity to extreme temperatures and sexual reproduction defects. Overall, our analysis of the three *P. anserina* mutations reveal for the first time the importance of the N-domain of a eukaryotic Lon protease.

## Results

### Identification of Three Mutant Alleles of the Mitochondrial Lon Protease Gene

**Figure 1:**
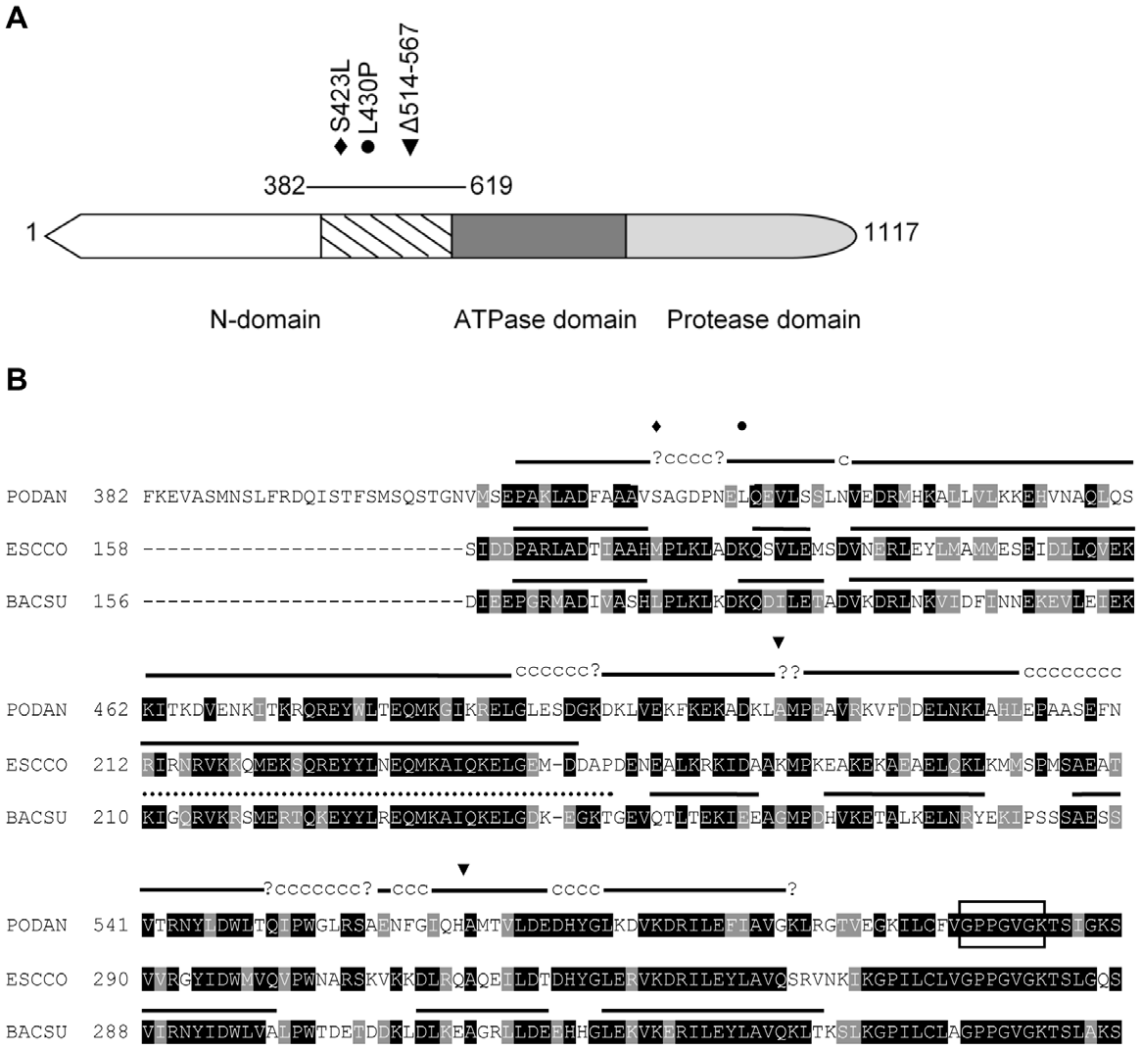
The mitochondrial PaLON1 protein. (A) Schematic representation of the PaLON1 protein outlining the three domains present in both prokaryotes and eukaryotes. The N-domain, which is the most divergent domain between Lon proteins, is followed by the highly conserved ATPase and protease domains. Within the N-domain, the most conserved region is within the C-terminal part (hatched). The line referring to residues 382 to 619 indicates the part of the protein presented in (B). Diamond (S423L), point (L430P), and inverted triangles (Δ514–567) mark changes induced by *PaLon1-31*, *PaLon1-1* and *PaLon1-f*, respectively. (B). Primary sequence and secondary structure of the C-terminal part of the N-domain of *B. subtilis*, *E. coli,* and *P. anserina* Lon proteases. Sequences were aligned using the Clustal W program. Conserved amino acids are boxed in black (identical) and gray (similar). For the *P. anserina* sequence (PODAN), changes induced by *PaLon1* mutations are represented by the same symbols as in (A). The GenBank accession numbers for *B. subtilis* (BACSU) and *E. coli* (ESCCO) proteins are CAA99540.1 and AAC36871.1, respectively. The Walker A motif of the central ATPase domain is boxed and begins at position 607, 356, and 354 in *P. anserina*, *E. coli* and *B. subtilis* proteins, respectively. The predicted consensus secondary structure of the PaLON1 region was determined on the NPS@ Web server [42] using a combination of available methods. For the same region, the secondary structure information available for *E. coli* and *B. subtilis* proteins ends at residue 245 [32] or contains a gap of 36 amino acids (dotted line), respectively [33]. For the *B. subtilis* protein, structure information was not available after the last α helix just before the Walker A motif. Secondary structures are indicated above each sequence as follows: lines, α helices; c letter, random coil (no secondary structure); and question mark (?), ambiguous state.

In a previous genetic screen, we isolated several independent *rgs* (resuming growth sector) mutations as suppressors of the *P. anserina* premature death syndrome [11]. Among the mutations, three were identified here as alleles of the nuclear *PaLon1* gene that encodes the mitochondrial Lon protease (Materials and Methods). *PaLon1* encodes an 1117 amino acid polypeptide that shares 31%, 44%, and 52% sequence identity with the *Escherichia coli* (La/Lon), *S. cerevisiae* (Pim1/Lon) and human (Lon) versions, respectively. While *PaLon1-31* and *PaLon1-1* contain missense mutations that lead to S423L and L430P amino acid substitutions, respectively, the *PaLon1-f* mutant has a 54 amino acid in frame deletion of residues 514 to 567. All three *PaLon1* mutations affect the N-domain of the protease (Figure 1A). The N-domain of the mitochondrial protein is longer than that of the bacterial Lon, as illustrated by the position of the conserved Walker A motif in the central ATPase domain (Figure 1B). The N-domain is also the most divergent region between eukaryotes and prokaryotes, although the C-terminal part of the N-domain sequence is well conserved, which is where all three mutations are located (Figure 1).

To analyze the defects associated with the *PaLon1* mutations and the null allele (see below), we used strains in a *mat*- context, unless otherwise stated.

### *PaLon1* deletion is viable and does not lead to mtDNA instability

To date, the consequences of deleting or disrupting the mitochondrial Lon protease gene has only been studied in two unicellular organisms, *S. cerevisiae* (*PIM1/LON*) and *Schizosaccharomyces pombe* (*lon1*), which differ in their tolerance to mtDNA instability [12] (and references therein). Inactivating the genes encoding the mitochondrial Lon protease in these two organisms does not result in the loss of viability and results in mtDNA deletion only in *S. cerevisiae* [7], [8], [13]. Deletion of the *PaLon1* gene (Δ*Lon1*) was performed by coding sequence (CDS) replacement with a hygromycin resistance cassette. *PaLon1* deletion did not prevent the ascospore germination process, which is critical to initiate the *P. anserina* life cycle. Thus, as in the case of *S. cerevisiae* and *S. pombe*, *PaLon1* is not an essential gene in *P. anserina*.

**Figure 2:**
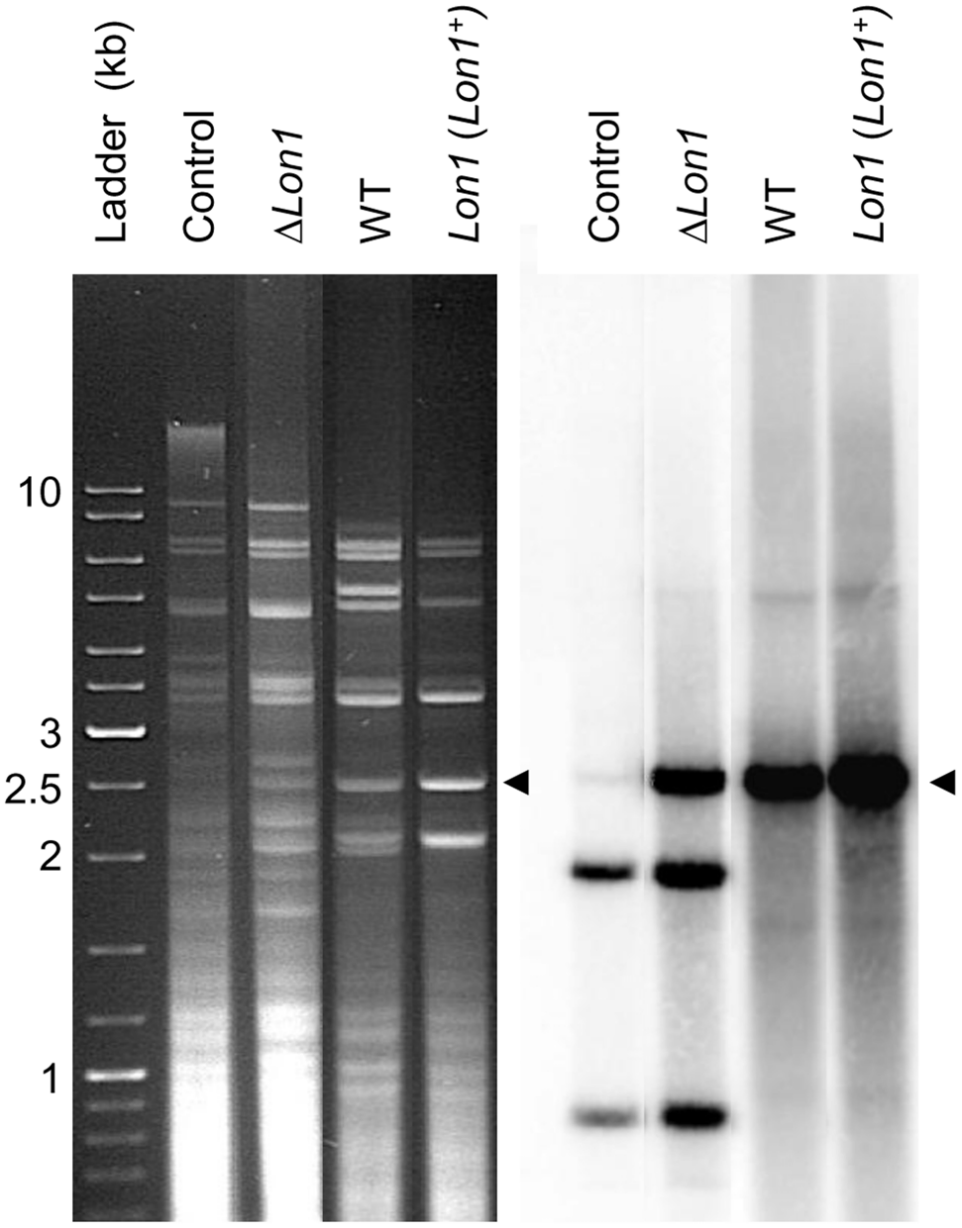
MtDNA profile of the Δ***Lon1*** **strain.** MtDNA profiles were determined by *Hae*III digestion of total DNA extracted from mycelium for the indicated dying strains. *Hae*III digestion of mtDNA from a young wild-type mycelium was used as a control. The arrows indicate the senDNAα multimeric subgenomic molecules present in dying strains (right and left panels). The identity of the senDNAα molecules was assessed by Southern blot analysis using an intron α specific probe (right panel). In addition to senDNAα (2.5 kb), hybridization revealed two *Hae*III fragments on the intact mitochondrial genome (1.9 and 0.8 kb). Note that these two bands were only detected in young wild-type (Control) and dying Δ*Lon1* strains.

We then examined mtDNA stability using specific-restriction profile analyses. An important feature of *P. anserina* is that cell death is always associated with mtDNA instability, which is characterized by variable mtDNA rearrangements and the disappearance of intact mitochondrial genomes. Amplification of a short mtDNA sequence (senDNAα) as circular multimeric subgenomic molecules is an additional marker of fungal death [14] (Figure 2). We observed that, at the time of death, Δ*Lon1* strains contained a small amount of senDNAα molecules; however, their mtDNA was unaltered, unlike in wild-type strains (Figure 2). Importantly, the Δ*Lon1* mutant displayed mtDNA instability and senDNAα amplification in the presence of a wild-type (*Lon1^+^*) transgene (Materials and Methods) (Figure 2). These results indicate that the cause of fungal death in the absence of PaLON1 is not mtDNA instability.

### Phenotypic characteristics of PaLon1 mutants

**Figure 3:**
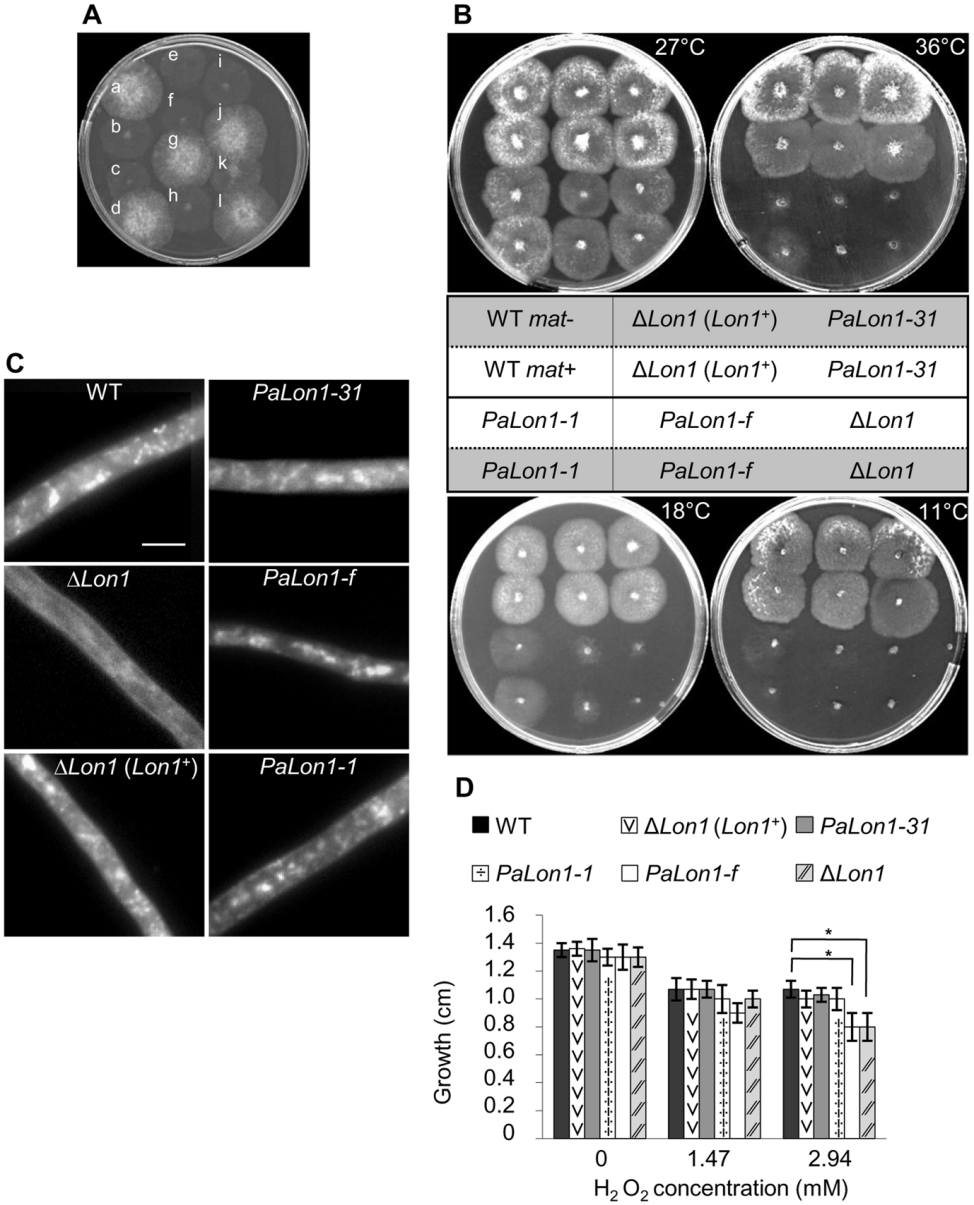
Phenotypic characteristics of *PaLon1* mutants. (A) Mycelium phenotype of *PaLon1-1* germinating ascospores on germination medium at 27°C after 2 days of growth. A cross between *PaLon1-1* and the wild-type strain gave rise to a progeny of *PaLon1-1* germinating ascospores with a less dense mycelium (letters: b, c, e, f, h, i, k) than that of the wild type (letters: a, d, g, j, l). (B) Growth phenotype exhibited by the *PaLon1* mutants. Strains were grown on M2 standard medium for 2 days (27°C and 36°C), 3 days (18°C), or 7 days (11°C). The genotype of each strain is shown in the table, except for the *rmp1-1* (*mat*−) and *rmp1-2* (*mat*+) alleles that are represented by a gray and white tone, respectively. (C) DASPMI staining of mitochondria. Mitochondria of growing strains (2 days at 27°C on M2) were stained with DASPMI, a vital mitochondrion-specific dye. For each indicated strain, filaments were gently mixed with a drop of DASPMI (25 mg/ml) directly on microscope slides and observed immediately with a fluorescence microscope (450–490/500–550 nm). All panels are at the same magnification and the scale bar corresponds to 5 μm. (D) Hydrogen peroxide (H_2_O_2_) sensitivity. Nine subcultures of each strain were inoculated in M2 or M2 supplemented with 0.005% (1.47 mM) or 0.01% (2.94 mM) H_2_O_2_. Growth (cm) was determined by measuring the radius of each thallus after 2 days at 27°C in the dark. Error bars indicate standard deviation. The statistically significant increase in the sensitivity of the *PaLon1*-f and Δ*Lon1* strains to 2.94 mM H_2_O_2_ is marked by an asterisk. In each case, the *p*-value (0.001) is below 0.05, as determined by the Mann-Whitney test.

As observed for the Δ*Lon1* ascospores, the *PaLon1-1*, *PaLon1-31* and *PaLon1-f* ascospores germinated. Nevertheless, mycelia arising from *PaLon1-1*, *PaLon1-f,* and Δ*Lon1* germinating ascospores on germination medium were less dense than that from wild-type ascospores (see Figure 3A for a comparison of *PaLon1-1* and wild-type ascospores). This defect was also observed on standard growth medium at the permissive temperature (27°C, Figure 3B). By contrast, germinating ascospores of the *PaLon1-31* mutant displayed no phenotypic changes, and mycelium growth was identical to that of wild-type *mat-* or *mat+* strains at 27°C (Figure 3B). Mitochondria of all strains grown at 27°C were stained with the vital mitochondrion-specific dye 2-(4-dimethylamino-styryl)-1-methylpyridium (DASPMI). While Δ*Lon1* mitochondria were thinner and showed reduced DASPMI staining, the mitochondria of the three other *PaLon1* mutants were similar to those of the wild type (Figure 3C). All Δ*Lon1* mitochondria defects were suppressed by the presence of the *Lon1^+^* transgene. To further characterize vegetative growth, we determined the life span of the *PaLon1* mutants by measuring the length (in cm) of maximal growth on standard medium at 27°C for several subcultures generated from germinating ascospores (Table 1). The Δ*Lon1*, *PaLon1-1*, and *PaLon1-f* strains displayed more heterogeneous and shorter life spans than wild-type *mat-* or *mat+* strains. The average life span of the *PaLon1-31 mat*+ mutant was similar (although more heterogeneous) to the wild-type *mat+* strain. By contrast, the life span of the *PaLon1-31 mat-* mutant was six times longer than that of the wild-type *mat-* strain. It has been previously shown that the *rmp1* gene, which is tightly linked to the *mat* locus, is involved in the death timing of several *P. anserina* mutants [15], [16], [17]. The *rmp1* gene is an essential gene that encodes a protein localized in the mitochondrial and/or cytosolic compartments [15]. This gene exists under two natural alleles, *rmp1-1* and *rmp1-2*, which are linked to the *mat*- and *mat*+ locus, respectively. Thus, we wanted to determine whether the *rmp1* gene is involved in the death timing of the *PaLon1-31* mutant. To address this question, we took advantage of the dominance of the *rmp1-1* allele over the *rmp1-2* allele, and constructed a *PaLon1-31 mat+ rmp1-2* strain expressing an *rmp1-1* transgene (Materials and Methods). The life span of this transgenic strain was over 27 cm and it showed no signs of death for the 23 subcultures tested. This indicates that the life span of the *PaLon1-31* mutant depends on the presence of the *rmp1-1* or *rmp1-2* allele (Table 1). However, unlike the other *PaLon1* mutations, the *PaLon1-31* mutation did not induce a decrease in fungal life span. In conclusion, the *PaLon1-1* and *PaLon1-f* mutants display phenotypic characteristics similar to those of the Δ*Lon1* mutant during ascospore germination and subsequent vegetative growth (including life span), whereas *PaLon1-31* does not.

**Table 1:** Life span of *PaLon1* mutants.

Genotype	*mat- rmp1-1*	*mat*+ *rmp1-2*
WT	9.7±0.7 (15)	10±0.4 (15)
Δ*Lon1*	4.1±2.1 (31)	3.7±1.9 (20)
Δ*Lon1* (*Lon1* ^+^)	9.2±0.8 (14)	10.2±0.6 (19)
*PaLon1-1*	6.3±3.6 (35)	3.5±1.7 (19)
*PaLon1-f*	4.2±2.2 (26)	3.9±1.3 (22)
*PaLon1-31*	60.2±38.1 (29)	11.4±5.9(23)

Values represent the mean life span measured in cm ± standard deviation. The numbers in parentheses show the number of subcultures tested for each indicated genotype.

Finally, we characterized the sexual reproduction of the *PaLon1* mutants. There were no defects in fructification development in female mutants fertilized by wild-type male gametes. Nevertheless, we observed a 1 day delay in asci production in *PaLon1-f* and Δ*Lon1* strains. We then estimated asci production efficiency by counting the number of asci ejected within 15 to 60 min, twice per day, for 4 days. For the *PaLon1-f*, *PaLon1-1*, and Δ*Lon1* strains, the overall asci production efficiency was 40% to 50% of that of the wild type. The *PaLon1-31* mutant was affected to a lesser degree, with an efficiency that was 70% of the wild-type level.

### Phenotype of the *PaLon1* mutants under stress conditions

*PaLon1* mutants were tested for their ability to grow at reduced (18°C and 11°C) and elevated (36°C) temperatures (Figure 3B). It should be noted that the growth difference between the wild-type *mat-* and *mat+* strains observed at 36°C depends on the nature of the *rmp1* allele [15]. At all temperatures, growth of the *PaLon1-31* mutant did not differ from that of the wild-type *mat-* or *mat+* strain. *PaLon1-1*, *PaLon1-f*, and Δ*Lon1* were similarly incapable of growing at 11°C, but showed differences at 18°C. Whereas the *PaLon1-f* and Δ*Lon1* strains displayed residual and no growth, respectively, the *PaLon1-1* mutant retained the ability to grow, albeit with an obvious defect. The *PaLon1-f* and Δ*Lon1* strains failed to grow at 36°C, while the growth of the *PaLon1-1* mutant was strongly altered in the presence of *rmp1-1* and was blocked in the *rmp1-2* context. In the presence of the *Lon1* ^+^ transgene, the Δ*Lon1* strain recovered its capacity to grow at 36°C, 18°C, and 11°C. These results indicate an absolute requirement for the Lon protease at high and low temperatures, whereas it is dispensable at the permissive temperature. *PaLon1* mutants were also tested for growth at 27°C in the presence of two concentrations of H_2_O_2_ (1.47 mM or 2.94 mM). As shown in Figure 3D, only the *PaLon1-f* and Δ*Lon1* strains were more sensitive to the highest concentration of H_2_O_2_ compared to the wild-type strain, and the H_2_O_2_ sensitivity of the Δ*Lon1* mutant was suppressed by the presence of the *Lon1* ^+^ transgene. The decrease in the H_2_O_2_ tolerance of the Δ*Lon1* strain is compatible with the finding that overexpression of *PaLon1* increases H_2_O_2_ tolerance [18].

Taken together, phenotypic analyses in normal physiological conditions (growth at 27°C and ascospore germination) revealed no differences between *PaLon1-1*, *PaLon1-f* and Δ*Lon1*. Nevertheless, the mutant containing an internal 54 amino acid in frame deletion (PaLON1-f) was more defective in its response to stress than the mutant with the L430P substitution (PaLON1-1). In addition, only the *PaLon1-f* mutant shared certain phenotypes with the Δ*Lon1* strain such as a similar sensitivity to high H_2_O_2_ concentrations and a delay in asci production. Finally, S423L (PaLON1-31) was shown to be the mutation with the least effect on the fungal life cycle and growth under stress conditions.

### The effect of *PaLon1* mutations on the steady state mRNA and protein levels

**Figure 4:**
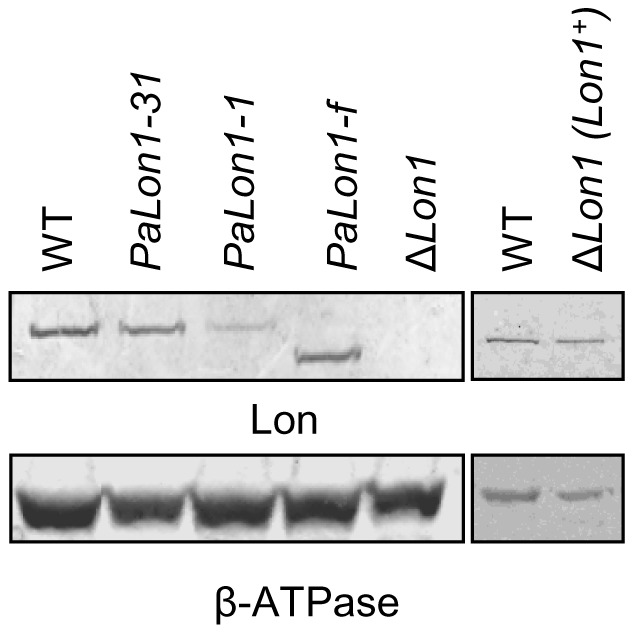
Detection of PaLON1 mutant proteins. Mitochondrial extracts (70 μg) purified from the indicated strains were resolved on an SDS-polyacrylamide gel and subjected to immunoblotting. The PaLON1 protein and the β-subunit of mitochondrial ATPase were detected by a *P. anserina* anti-PaLON antibody and an *S. cerevisiae* anti-Atp2 antibody, respectively. Mitochondrial extraction and western blotting were repeated at least twice for each strain. The left and right panels correspond to two independent membranes.

To further characterize the *PaLon1* mutants, we analyzed *PaLon1* gene and protein expression by RT-qPCR and western blot analyses, respectively (Materials and Methods). The abundance of the *PaLon1* transcript was similar in all strains, as revealed by mutant to wild-type ratios of 1.090±0.3, 1.382±0.4 and 1.077±0.3 for the *PaLon1-1*, *PaLon1-f* and *PaLon1-31* strains, respectively. For western blot analyses, the same amount of purified mitochondria was loaded onto gels and the PaLON1 protein level was normalized using βATPase as a loading control (Figure 4). As expected, we observed no signal at the predicted size of the PaLON1 protein (118 kD) in the mitochondrial extract from the Δ*Lon1* strain. Furthermore, no change in Δ*Lon1* mitochondrial content was observed for the inner membrane protein βATPase (Figure 4) or the outer membrane protein porin (data not shown). We also detected equivalent amounts of the PaLON1 protein when comparing the expression of the *PaLon1* gene from its own locus (WT) versus that from an ectopic locus (Δ*Lon1* (*Lon1^+^*) strain). The internal 54 amino acid in frame deletion of the PaLON1-f polypeptide gave rise to a smaller immunoreactive species, whose level was not significantly different from that of the wild type PaLON1 protein. The protein level of PaLON1-31 (S423L) was only slightly reduced, whereas that of PaLON1-1 (L430P) was reduced by a factor of 4.8. The proline residue may result in modified PaLON1 folding and hence, could change the susceptibility of the mutant protein to degradation. By contrast, the internal deletion had no effect on the protein level, which suggests that the 54 deleted residues may define a domain that is independently folded.

### Effect of the *PaLon1* mutations on mitochondrial proteolytic activity

To determine whether the proteolytic activity of *PaLon1-31*, *PaLon1-f*, and *PaLon1-1* differed from that of the wild type, ATP-dependent proteolytic activity of wild-type and mutant mitochondrial extracts was quantified by examining FITC-casein degradation. Reactions were performed in the presence of ATP using the same amount of mitochondrial extracts from each strain. The proteolytic activity of each mutant strain was compared with that of the wild type and the resulting ratios of mutant to wild-type proteolytic activity were used to evaluate the effects of the *PaLon1* mutations. The lowest proteolytic activity (∼40% that of the wild type) was observed for mitochondrial extracts from the *PaLon1-31* strain, which indicates that the PaLON1-31 protein is defective in the degradation of the FITC-casein substrate. By contrast, the proteolytic activity of mitochondrial extracts from the *PaLon1-f* strain was similar to that of the wild type, with the exception of a decrease in activity after 5 h of incubation. The proteolytic activity of mitochondrial extracts from the *PaLon1-1* strain was ∼70% of that of the wild type. Our observation that the PaLON1-1 protein was 4.8-fold less abundant than wild type suggests that the proteolytic activity of the PaLON1-1 protein was not strongly affected (Table 2).

**Table 2:** Mitochondrial proteolytic activity of *PaLon1* mutants.

	Genotype		
Time	*PaLon1-31*	*PaLon1-1*	*PaLon1-f*
3 h	0.43±0.2	0.69±0.1	1.18±0.06
4 h	0.45±0.02	0.69±0.09	1.29±0.39
5 h	0.41±0.03	0.6±0.11	0.68±0.04

Values represent the average ratio of mutant/wild type ± standard deviation of three independent experiments. Three incubation times were used for each experiment.

## Discussion

In this study, we demonstrated that the absence of the mitochondrial Lon protease is tolerated in *P. anserina,* but leads to a decrease in life span by a factor of 2.5, while overexpression of the *PaLon1* gene was previously reported to increase life span by a factor of 1.7 [18]. Absence of PaLON1 leads to defects in ascospore germination and sexual reproduction, two fungal developmental steps that were recently showed to be finely regulated [19]. This suggests that Lon regulates specific functions during the fungal life cycle, potentially by controlling the temporal availability of specific proteins. We found that PaLON1 is indispensable at low and high temperatures, which indicates that no other mitochondrial ATP-dependent proteases, specifically ClpXP, can compensate for the absence of Lon under these conditions. Notably, no phenotypic defects were reported for a *P. anserina* strain lacking *ClpP* [18]. Loss of the *PaLon1* gene is also associated with a weak vital staining of Δ*Lon1* mitochondria, which is indicative of a decrease in membrane potential, and was also observed in an *S. pombe Lon* deletion strain [13] and human *Lon* down-regulated cells [20]. Because the three other *PaLon1* mutants showed mitochondrial staining similar to that of the wild type, the absence of the PaLON1 protein is likely responsible for the membrane potential alteration in the Δ*Lon1* strain. This may be related to the chaperone-like activity of Lon, which has been shown to assist in the assembly of inner membrane complexes in yeast *S. cerevisiae* and mammals [21], [22]. In contrast to the wild type, death of the *P. anserina* Δ*Lon1* strain is not associated with mtDNA instability, suggesting a more efficient reparation process in this strain. This mechanism was previously proposed to explain why human Lon-depleted cells exhibited less mtDNA damage than control cells under H_2_O_2_ stress [23]. Since death of the Δ*Lon1* strain cannot be attributed to mtDNA instability, we suggest that the absence of Lon protection against the accumulation of defective mitochondrial proteins accelerates fungal death.

Lon is an allosteric enzyme in which fixation of the substrate occurs on a site distinct from the catalytic site [24], [25]. It was proposed that efficient degradation only occurs for high affinity substrates that bind at two distinct allosteric sites. ATP hydrolysis then promotes a conformational change that allows the capture and translocation of the substrate to the protease domain [25]. Lon proteolysis efficiency was recently found to be modulated by the degradation tags of the substrates themselves, revealing a complex relationship between Lon and its substrates [26]. Consequently, impairment in substrate degradation can result from the alteration of several steps, including substrate binding.

In vitro assays using *P. anserina* mitochondrial extracts strongly suggest that the PaLON1-1 (L430P) and PaLON1-f (Δ514-567) proteins are not severely defective in FITC-casein degradation. This indicates that the protease activity of PaLON1-1 and PaLON1-f mutant proteins are intact. However, the *PaLon1-1* strain displays defects that are either identical to or more attenuated than the Δ*Lon1* strain. The primary defect of the *PaLon1-1* mutant is most likely the level of the PaLON1 protein, which was 4.8-fold lower in the mutant than in the wild type. These results indicate that Lon is a limiting factor for the fungal life cycle. Compared to *PaLon1-1*, the *PaLon1-f* strain is phenotypically identical to the Δ*Lon1* strain, especially during development and under stress, and yet we detected no decrease in the PaLON1 protein level. The PaLON1-f protein, despite being efficient for the degradation of an unfolded substrate (FITC-casein) in vitro, may exhibit in vivo defects in the degradation of partially unstructured substrates (stress conditions) and some native proteins that are temporally required during development. Thus, we propose that the internal deletion of PaLON1-f affects substrate binding efficiency, which is critical for cell response to stress and for fungal development.

The FITC-casein degradation defect in *PaLon1-31* mitochondrial extracts is clearly not due to an inactivation of the protease activity, since the *PaLon1-31* strain does not exhibit the same defects as the Δ*Lon1* strain. The most likely explanation is that the PaLON1-31 protein has a higher affinity for substrates present in mitochondrial extracts than for FITC-casein. This hypothesis implies that natural substrates and FITC-casein share at least a common allosteric binding region, and that the capture of natural substrates is more efficient for the PaLON1-31 mutant protein than for the wild type. Such an increase in substrate binding efficiency may explain why the *PaLon1-31* strain does not display a phenotype during fungal development and under stress conditions, and results in the distinctive life span phenotype observed.

The three *P. anserina* mutations uncovered in this study reveal for the first time the importance of the N-domain of a eukaryotic Lon protease, which leads us to propose that the N-domain of mitochondrial Lon protease is implicated in substrate binding. Substrate interaction by the N-domain was also proposed for bacterial Lon, but was inferred from the results of experiments measuring changes in α-casein degradation in vitro with various purified N-terminal Lon truncations [27], [28], [29]. Interestingly, a single amino acid change in the N-domain of *E. coli* Lon protease was shown to affect substrate specificity in vivo. The *E. coli* E240K mutant behaves phenotypically like the Δ*Lon* strain (stabilization of the substrate) for certain substrates, while it behaves like the wild-type (degradation of the substrate) for others [30]. While the structure of a full-length Lon is not yet available, the structure of the N-domain has been resolved in bacteria; it consists of two subdomains, a β-sheet rich, globular N-terminal part, and an all-helical C-terminal part [31], [32], [33]. *PaLon1* mutations are all located in the C-terminal part of the N-domain, as is the *E. coli* E240K substitution. The glutamate residue is strictly conserved (position 490 in PaLON1) and, in bacteria, belongs to a putative coiled coil domain (predicted by the program COILS) [34] that was proposed to play a role in oligomeric structure and substrate binding [28], [30], [33], [35]. However, no such coiled coil structural motif is predicted for the C-terminal part of the Podospora PaLON1 protein (according to the program COILS); however, we found that the region could adopt a secondary structure that correlates with structural data from bacterial proteins indicating a region solely composed of α helices for which the number, length, and position are conserved between Podospora and bacteria (Figure 1B). We therefore propose that it is the highly conserved α helical arrangement of the C-terminal part of the N-domain of Lon that participates in substrate binding in both prokaryotes and eukaryotes.

## Materials and Methods

### *P. Anserina* Strains, Growth Conditions, and Transformation Experiments

All strains used in this study were derived from the S wild-type strain to ensure a homogeneous genetic background. Details on standard culture conditions, media, protoplast formation, protoplast transformation, and genetic methods are available at http://podospora.igmors.u-psud.fr. The standard growth medium and ascospore germination medium used were M2 and G, respectively. When necessary, hygromycin (Euromedex), nourseothricin (Werner BioAgents) or phleomycin (Euromedex) was added at a concentration of 100, 50, and 10 µg.ml^−1^, respectively.

### Identification of the rgs Mutations

Three *rgs* mutations (*rgs1*, *rgs31* and *rgsf*) were localized to the long arm of chromosome III. The *rgs1* mutation was mapped by positional cloning using the following associated defects: alteration during ascospore germination on G medium and the absence of growth at a low temperature on M2 medium. For positional cloning, we used the progeny of a cross between an S strain and a T strain (TS24 strain), since many molecular polymorphic markers have been characterized for the two geographically distinct S and T strains [36]. The fertile TS24 (*rgs1^+^*) strain retained 11 microsatellites markers that are characteristic of the T strain on the long arm of the chromosome III. After a cross between the *rgs1^+^* (TS24) strain and the *rgs1* (S origin) mutant, the phenotype of strains from 187 ascospores were determined. Among them, 73 *rgs1^+^* strains (i.e., no defect during ascospore germination and growth at 11°C) were genotyped by PCR analysis, which used a set of 11 primer pairs (nucleotide sequences are available on request) to determine the S or T origin of the amplified region. This global analysis localized the *rgs1* gene to a region of 240 kb. To further narrow down the region, four informative strains were genotyped with four new polymorphic microsatellite markers. Vectors that contained wild-type genomic inserts covering the further restricted 98-kb region were used to transform *rgs1* mutant protoplasts. This led to the identification of a complementing region of about 10 kb including the Pa_3_4170 CDS, which was shown to be responsible of the complementation. For this, the Pa_3_4170 CDS was amplified as a 4.8 kb DNA fragment from the complementing vector using primers 5′KpnPIM (atggtaccaaaggtgagacgccagtg) and 3′SalPIM (caaacgtcgaccctctctctcatcacac) that harbor *Kpn*I and *Sal*I restriction sites, respectively. The PCR product was cloned at the corresponding sites into the PCB1004 vector, which contains a hygromycin resistance cassette. The resulting pCBPIMKS plasmid was used for complementation assays after sequencing Pa_3_4170 CDS and its 5′ and 3′ flanking regions over 1050 bp and 471 bp, respectively. The *rgs31* mutation was similarly mapped by positional cloning, but in this case, we used a leaky growth phenotype at 11°C on M2 medium that was only observed when ascopores were previously maintained for an extensive time (1 month) at 4°C on G medium. The *rgsf* mutation was mapped by a complementation test, which was performed between the *rgsf* and *rgs1* mutants using the recessive phenotype they share during ascospore germination. The Pa_3_4170 CDS encodes the mitochondrial Lon protease. Due to the presence of a putative peroxisomal Lon protease gene (Pa_6_2970 CDS) in the *P. anserina* genome, we named the mitochondrial Lon protease gene, *PaLon1* and the *rgs1*, *rgs31* and *rgsf* mutants were renamed *PaLon1-1*, *PaLon1-31* and *PaLon1-f*, respectively. Finally, all three mutations were characterized at the nucleotide level by direct sequencing of the *PaLon1* CDS and its 5′ and 3 flanking regions over 585 bp and 363 bp, respectively. Double strand sequencing was performed on genomic DNA purified from each strain.

### Deletion of the *PaLon1* gene (Δ*Lon1*)

To delete the *PaLon1* CDS, its 5′ (1014 bp) and 3′ (1046 bp) flanking regions were first amplified by PCR from wild-type genomic DNA using primer pairs 5′KpnPIM (atggtaccaaaggtgagacgccagtg)/5′R1AscI (gctggcgcgccagcattgtgg) and 3′R1AscI (ggaggcgcgccattaggtagatgttgggag)/3′PIMextR (gtgggcagggtcactcaatg). PCR fragments were digested by *Kpn*I/*Asc*I and *Asc*I, respectively, and inserted between the *Kpn*I and *Eco*RV sites of a Bluescript plasmid. The resulting plasmid was linearized at *Asc*I to insert a hygromycin resistance (hygro^R^) cassette. The final construction results in a hygromycin cassette flanked by 5′ (1014 bp) and 3′ (1046 bp) borders of *PaLon1* CDS. This cassette was PCR amplified using primer pair LON 5′(gagcgtccctgttgaactag)/3′PIMR (gcgttgcatccagctctctg) and was used to transform protoplasts of a Δ*PaKu70* mutant strain, which increases homologous integration events [37]. Correct gene replacement of the *PaLon1* CDS by the hygromycin cassette was verified by PCR and Southern blot analyses. The Δ*PaKu70* allele was segregated after crossing with the wild type to obtain a Δ*Lon1* strain.

### Construction of a *PaLon1* transgene (*Lon1* ^+^)

The 4932 bp *Sal*I-*Pvu*II fragment of the pCBPIMKS plasmid was subcloned in the compatible *Eco*RV-*Sal*I sites of a pAPI508 vector that contains a nourseothricin resistance cassette [37]. The resulting plasmid was used to transform wild-type protoplasts. Nourseothricin resistant transformants (nour^R^) were crossed with the Δ*Lon1* hygromycin resistant (hygro^R^) strain and nour^R^/hygro^R^ progeny were assessed for wild-type phenotype. One Δ*Lon1* (*Lon1^+^*) strain was used for all subsequent experiments.

### Construction of *PaLon1-31* strains expressing the *rmp1-1* and *rmp1-2* alleles

A *PaLon1* ^+^ *rmp1-2 mat+* strain expressing a functional *rmp1-1* transgene (*rmp1-1*) associated with a phleomycin-resistance cassette [15] was crossed with a *PaLon1-1 rmp1-1 mat-* strain. As the *rmp1-1* transgene segregates independently of the *PaLon1* gene, we isolated *PaLon1-1 rmp1-1* (*rmp1-1*) *mat-* strains, which displayed characteristics identical to the *PaLon1-1 rmp1-1 mat-* strains. A *PaLon1-1 mat-* strain expressing two copies of the *rmp1-1* allele was crossed with a *PaLon1-31 rmp1-2 mat+* strain. Among the progeny, we obtained *PaLon1-31 rmp1-2* (*rmp1-1*) *mat+* strains.

### Rapid DNA extraction used for strain genotyping

After 2 days of growth at 27°C on standard medium covered with cellophane layers, mycelium was recovered and transferred to a 2-ml microfuge tube. Glass beads and 20 μl of a fresh 0.5 M NaOH solution were added and the mycelium was ground for 40 s at 5 m/s using the FastPrep FP 120 Cell Disrupter Instrument (Qbiogene). Tubes were then incubated for 30 s in boiling water and cooled in ice. Afterwards, 100 μl of a solution containing 1 M Tris pH 8 and TE pH 8 (1∶4 v/v) were added and 1 μl of the extract was used for each PCR amplification.

### Rapid DNA extraction used for mtDNA profile analyses

Total DNA from dying strains was extracted by the rapid method described in [38], with the following modifications: the mycelium was frozen in liquid nitrogen in extraction buffer and stored at −20°C. After thawing, the mycelium was ground twice with the FastPrep FP 120 Cell Disrupter Instrument (Qbiogene) for 30 s at 5 m/s. After DNA extraction, the first DNA pellet was resuspended in NaOAc 0.3 M pH 6 and precipitated by ethanol. The mtDNA profile was revealed by *Hae*III digestion. After electrophoresis, Southern blot analysis was performed using standard conditions and a ^32^P-labeled specific probe corresponding to intron α of the mitochondrial COI gene encoding the cytochrome oxydase subunit I.

### Analyses of *PaLon1* gene expression by real-time quantitative PCR

Total RNA was extracted from mycelium collected after 2 days of growth at 27°C on standard medium covered with cellophane layers. RT-qPCR experiments were performed with 4 µg of total RNA [19]. Four biological replicates of each strain were examined. To ensure specific detection of *PaLon1* cDNA with minimal interference from genomic DNA, we designed one primer positioned on two consecutive exons and checked for the absence of contaminating genomic DNA by performing PCR and qPCR on non-reverse transcribed RNAs. *PaLon1* cDNA was amplified using the forward primer 5′ GTTGGAGTTGCCTGAG/AATATC 3′ (slash indicates the position of the intron in the genomic DNA) and the reverse primer 5′ CTACCCATCTAATGACCAAATC 3′. Amplification gave rise to a product of 287 bp. Three reference genes were used for normalization [39]: *GPD* (glyceraldehyde 3-phosphate deshydrogenase, formally Pa_3_5110), *PDF2* (protein phosphatase PP2A regulatory subunit A, Pa_7_6690) and *TIP* (type 2A phosphatase activator TIP41, Pa_7_8490). For each primer pair, efficiency was determined by making serial dilutions of a mixture of all RT samples, as recommended by MIQE guidelines [40]. Efficiencies were between 1.87 and 1.98. All PCR products exhibited melting curves indicating the amplification of a single product. RT-qPCR normalization, standard error computation, expression level calculation and statistical analyses were performed with REST-MCS [41].

### Mitochondria purification

Mitochondria were isolated from mycelium collected after 2 days of growth at 27°C on standard medium covered with cellophane layers. Mycelium was dried, frozen, and placed into a pre-cooled Teflon shaking flask with two grinding stainless steel balls. Flasks were then placed in a Mikro-Dismembrator machine (Sartorius) and after 1 min of shaking at a frequency of 2,600 per min, the mycelium was crushed into a fine powder. This was then resuspended in 0.7 M sorbitol buffer (0.7 M sorbitol, 50 mM Tris-HCl, pH 7.5, 0.2 mM EDTA) with protease inhibitor cocktail (Roche Diagnostics GmbH, Mannheim, Germany) and the mixture was clarified by centrifugation at 3,500 rpm (low speed) for 10 min at 4°C. The supernatant was recovered and centrifuged at 13,000 rpm (high speed) for 20 min at 4°C to sediment mitochondria. This first mitochondrial pellet was resuspended in 0.7 M sorbitol buffer and rinsed by centrifugation at low speed. The supernatant was then re-centrifuged at high speed to sediment purified mitochondria. The mitochondrial pellet was finally resuspended in 0.7 M sorbitol buffer and the mitochondrial protein concentration was determined using the Bio-Rad assay.

### Western blotting

Mitochondrial extracts (70 μg) were resolved on Pre-Cast gels (NuPAGE 7% Tris-Acetate gels, Invitrogen), transferred onto membranes and probed with the following antisera: *S. cerevisiae* anti-βATPase (Atp2p) antibody (used at 1/50000), kindly provided by J. Velours and G. Dujardin, and *P. anserina* anti-PaLON and anti-PORIN antibodies (used at 1/1000 and 1/5000, respectively), both kindly provided by H. Osiewacz. The binding of primary antibodies was either revealed by incubation with a peroxidase-conjugated secondary antibody (anti-rabbit, Promega) and chemiluminescent ECL reagent (GE Healthcare) or by incubation with an alkaline phosphatase-conjugated secondary antibody and BCIP/NBT colorimetric detection system (Promega). Quantification of immunoreactive species was carried out with the ImageJ 1.43 software (Windows version of NIH Image, http://rsb.info.nih.gov/nih-image/).

### Protease activity

To determine proteolytic activities, we used a protease fluorescent detection kit (PF0100, Sigma) according to the manufacturer’s instructions. Mitochondrial extracts (70 μg) were incubated with the FITC-casein substrate, incubation buffer and 8 mM ATP at 37°C in the dark. For each mitochondrial extract tested, an equal aliquot was collected after 3, 4 or 5 h of incubation and proteins were precipitated by 0.6 N TCA for 30 min at 37°C in the dark. After centrifugation at 10,000 g for 10 min, 10 μl of each supernatant was mixed with 1 ml of assay buffer and 250 μl were transferred to a black 96-well plate (Sigma). Fluorescence was measured with excitation/emission wavelengths of 485/535 nm in a multiplate reader (Chameleon). Activities were expressed as fluorescence units (FU).
